# Personalized medicine for locally advanced rectal cancer: five years of complete clinical response after neoadjuvant radiochemotherapy—a case report with a literature review

**DOI:** 10.3389/fsurg.2024.1385378

**Published:** 2024-03-25

**Authors:** Dennis Obonyo, Verena Uslar, Dirk Weyhe, Navid Tabriz

**Affiliations:** Carl von Ossietzky University Oldenburg, University Clinic for Visceral Surgery, Pius-Hospital Oldenburg, Oldenburg, Germany

**Keywords:** rectal cancer, neoadjuvant radiochemotherapy (nRCT), complete clinical response (cCR), non-operative management (NOM), watch-and-wait (W&W) strategy, personalized medicine, case report

## Abstract

We present a case report of a 73-year-old male patient with a complete clinical response following neoadjuvant radiochemotherapy of mid-rectal adenocarcinoma. The patient was initially diagnosed with stage IIIB microsatellite stable mid-rectal adenocarcinoma in February 2017. During restaging in June 2017, which included rectoscopy, endosonography, computed tomography and magnetic resonance imaging, a complete clinical response was observed. After appropriate consultation, a watch-and-wait strategy was chosen. During stringent follow-up every 3 months for the first 3 years and thereafter every 6 months, no recurrence or regrowth was observed. After the fifth year of complete clinical response, we recommended an annual follow-up. As of November 2023, the patient has no signs of recurrence or late toxicity after radiochemotherapy. The omission of resection in patients with locally advanced rectal cancer and the establishment of a watch-and-wait strategy are currently under discussion as possible treatment courses in patients with complete clinical response. Long-term data on watch-and-wait strategies for patients with a complete clinical response in locally advanced rectal cancer are rare. A clear national and international accepted standardization of follow-up programs for patients managed by a watch-and-wait strategy in the long-term is missing. Here, we report the case of a patient who had undergone a follow-up program for more than five years and discuss the current literature. Our case report and literature review highlights that a watch-and-wait strategy does not seem to increase the risk of systemic disease or compromise survival outcomes in selected locally advanced rectal cancer patients. Thus, our case contributes to the growing body of knowledge on personalized and precision medicine for rectal cancer.

## Introduction

The standard therapy for patients with locally advanced rectal cancer (LARC) is neoadjuvant radiochemotherapy (nRCT) followed by total mesorectal excision (TME), with or without postoperative chemotherapy (1–3). Up to one-third of the patients receiving nRCT for LARC achieve a complete clinical response (cCR) and/or a pathologic complete remission (pCR) (4–6). Habr-Gama and colleagues reported several series in which the cCR rate ranged from 26% to 38% (7–10). Thus, the acceptance of non-operative management (NOM) or organ preservation for LARC patients via the watch-and-wait (W&W) strategy (4, 6, 7, 9) is increasing. Owing to the fact that the TME is associated with a risk of surgery-related complications, morbidities and mortality (11, 12), there are quite a number of patients who decline abdominoperineal resection, or a Hartmann procedure with permanent colostomy or even low anterior rectal resection without creation of protective ileostomy or colostomy. Compared with the TME, the W&W strategy achieves similar overall survival and better preservation of organ anatomy and physiological function. Meta-analyses studying the W&W strategy vs. the TME indicate that the W&W group has a greater local recurrence rate than the TME group does, but the overall survival and rate of distant metastasis are similar between the two groups (13–15). Furthermore, Zhang et al. showed that elevated carcinoembryonic antigen (CEA) levels ≥5 ng/ml after chemoradiotherapy is negatively associated with tumor response to total neoadjuvant therapy (TNT) (16). Therefore, through consistent and standardized follow-up examinations, NOM with the W&W strategy can achieve equivalent results in patients with cCR compared to those with TME. Recommendations for a stringent course of investigation during follow-up with regard to the method and time point never existed at the first presentation in 2017 in many national guidelines.

Here, we present a case of cCR in a patient with LARC in the midrectum after nRCT with more than 5 years surveillance via the W&W strategy and provide recommendations for follow-up management in patients with cCR after nRCT in LARC, as this approach is feasible and safe for appropriately selected patients. This highlights the need for precision personalized medicine in rectal cancer patients.

## Case presentation

**Figure 1:**
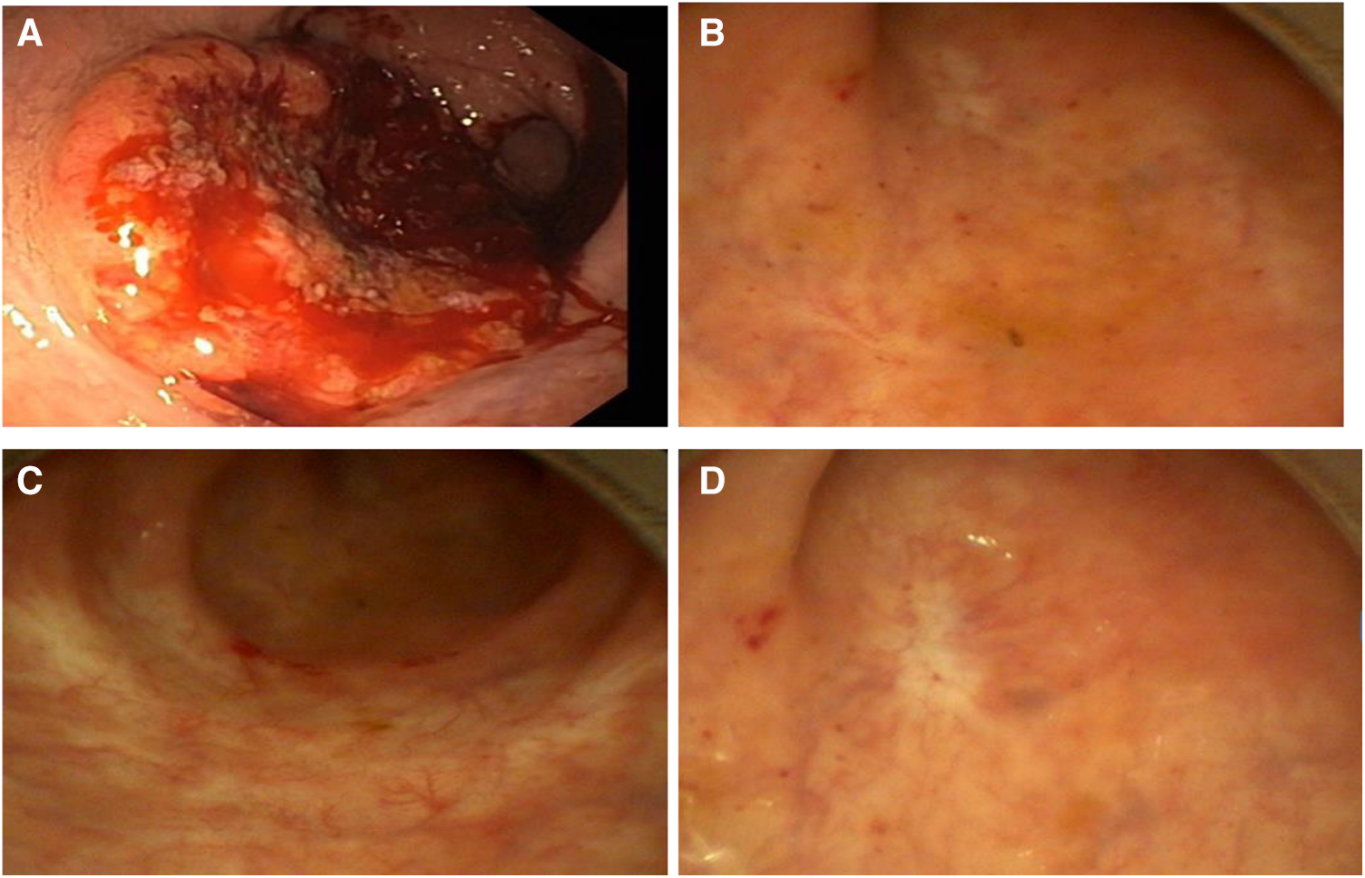
Colonoscopy/rectoscopy images; (**A**) February 2017, (**B**) May 2019, (**C**) May 2021 and (**D**) November 2023.

**Figure 2:**
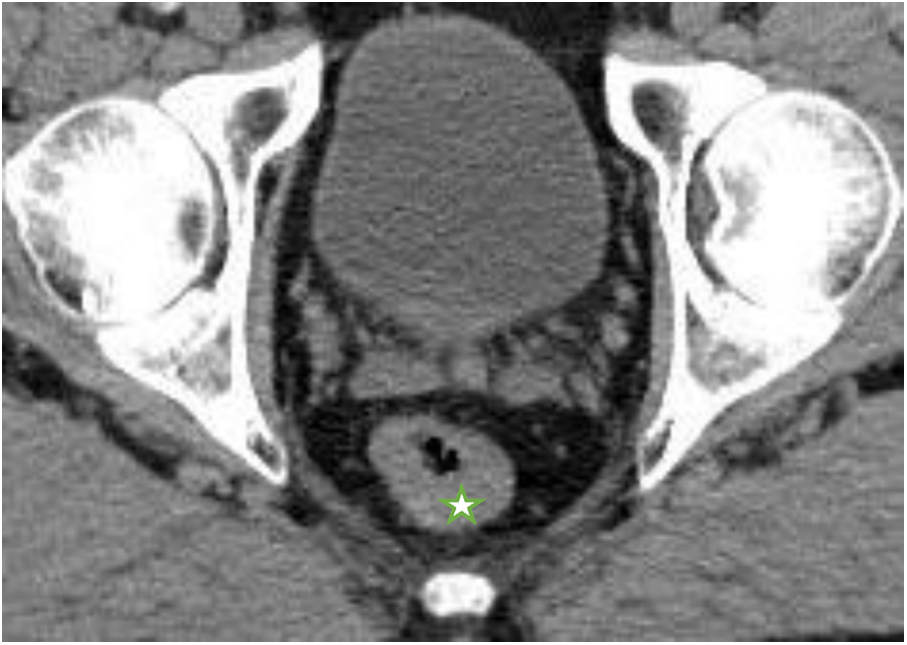
Computed tomography (February 2017) showing wall thickening of the rectum, marked with a star.

**Figure 3:**
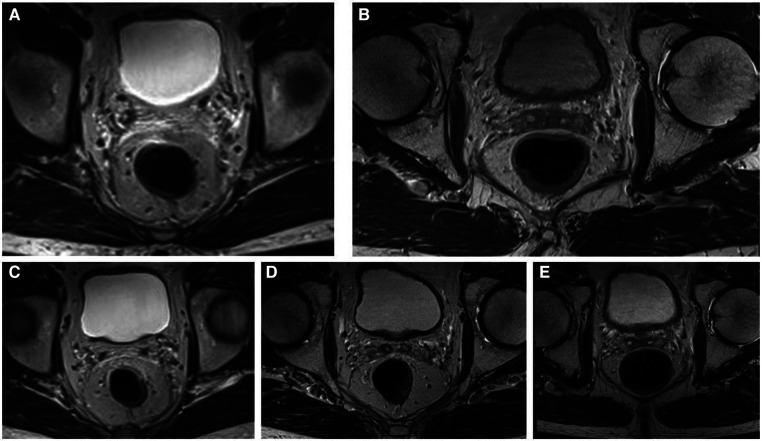
Dotarem-enhanced T2 magnetic resonance (MR) images of the patient during the W&W follow-up visit with no signs of local regrowth or lymph node metastasis; (**A**) June 2017, (**B**) June 2018, (**C**) May 2019, (**D**) June 2020, and (**E**) May 2021.

A 79-year-old male German patient (i.e., 73 years old at first presentation) was diagnosed with microsatellite stable mid-rectaladenocarcinoma during a screening colonoscopy without any clinical symptoms in February 2017 (see also Table 1). The patient had no relevant comorbidities and was in good clinical condition. Colonoscopy revealed a semicircular and exophytic tumor with a size of 50 mm in the rectum 8 cm from the anal verge (Figure 1). Pathology of a biopsy specimen revealed moderately differentiated adenocarcinoma of the colorectal type. Abdominopelvic computed tomography (CT; Figure 2) and pelvic magnetic resonance imaging (MRI) showed concentric growing rectal carcinoma with locoregional lymph node metastasis in the mesorectum as well as circumferential wall thickening with perirectal fat infiltration (Figure 3A). No distant metastases were found. Endoscopy revealed a tumor with a maximum thickness of 13 mm in the midrectum that broadly exceeded the muscularis, as well as suspicious regional lymph nodes. The tumor marker carcinoembryonic antigen (CEA) level was within the normal range. The clinical stage was determined to be uT3uN1cM0; stage IIIB according to the Union for International Cancer Control (UICC) staging manual (7th edition).

**Table 1:** Timeline.

February 2017	Incidental diagnosis by screening colonoscopy
	Rectoscopy, abdominopelvic CT, MRI, CT scan of the chest endosonography, CEA
February 2017	Discussion in tumor board and recommendation of nRCT
March 2017	Start of neoadjuvant therapy with up to 50.4 Gy radiotherapy and simultaneous chemotherapy with capecitabine 825 mg/m^2^
May 2017	End of neoadjuvant therapy
June 2017	Restaging including digital rectal examination, rectoscopy, abdominopelvic CT, MRI, endosonography with biopsy showing only a fibrotic mass with no viable tumor cells
September 2017–June 2022	Stringent follow-up including digital rectal examination, rectoscopy, measurement of tumor markers CEA, chest radiology, abdominopelvic sonography and MRI

The case was then discussed by our multidisciplinary tumor board, for which nRCT with up to 50.4 Gy radiotherapy and simultaneous chemotherapy with capecitabine 825 mg/m^2^ twice daily were recommended. Neoadjuvant therapy started in March 2017, and was given for six weeks without interruption or absence of any severe complications. Briefly, the total dose of preoperative radiotherapy was 50.4 Gy, which was given in a fractionated manner over a period of 6 weeks (1.8 Gy × 28 fr over 6 weeks) in the supine position. The clinical target volumes included the gross mural tumor, regional lymph nodes in the mesorectum and presacral space and the internal iliac and distal common iliac lymphatics. The oral concurrent chemotherapy with capecitabine (825 mg/m^2^) was administered twice daily.

A reevaluation of the nRCT response and simultaneous planning of the TME and a protective ileostomy were scheduled approximately 8 weeks after nRCT completion. On presentation in June 2017, no tumor mass or stenosis was palpable during a digital rectal examination. A slight bluish venous dilatation with negligible ulceration was evident on rectoscopy. On endosonography no tumor or lymph nodes were observed. A wall thickening of the rectum was described. MRI revealed no definite mass lesion but mucosal thickening of the rectum after nRCT and no evidence of metastatic lymph nodes. These findings were discussed in detail with the patient and his relatives. We explained the extent of surgical therapy with TME and the possibility of protective ileostomy. The patient refused to undergo surgery. Therefore, we proposed a follow-up regimen for the patient, including digital rectal examination combined with rectoscopy (through an experienced colorectal surgeon), CEA measurement, chest radiology, abdominal ultrasound and pelvic MRI every 3 months. The advantages and disadvantages of these methods were discussed in depth. A written informed consent was obtained from the patient.

At the end of August 2017, there was no tumor seen during rectoscopy, and the bluish venous dilatation with negligible ulceration had disappeared. A wall thickening was suspected at 8 cm from the anal verge. This was confirmed by endosonography. We opted to carry out a biopsy at the suspected area. The final histological findings showed only a fibrotic mass without tumor cells. All other examinations, including pelvic MRI, abdominal ultrasound and chest radiology, showed no evidence of local or lymph node recurrence or distant tumor manifestation. The tumor marker CEA was also within the normal range. Follow-up evaluations were performed every three months for the first three years until March 2020, and no regrowth or evidence of lymph node recurrence or distant tumor manifestation was observed. No further thickening of the rectum was observed 12 months after the nRCT. Thereafter, we extended the time interval between follow-up appointments to 6 months. To date, after more than 5 years of follow-up, no evidence of regrowth or recurrence has been observed. We recommended an annual follow-up examination as well as a colonoscopy to rule out a second carcinoma.

## Discussion

With this case report, we are able to provide additional evidence that NOM via the W&W strategy can be feasible and safe. Furthermore, surgery can be possibly avoided for patients with cCR after nRCT in locally advanced rectal cancer when a structured follow-up evaluation is implemented. NOM with the W&W strategy has gained popularity for patients with cCR after nRCT following LARC. This forces us, as the involved physicians, to resort to recommendations that the national guidelines do not provide. On the other hand, the increasing interest in NOM with the W&W strategy requires reliable methods to identify patients with cCR (17). We defined cCR as follows: endoscopy showing only a white scar with or without telangiectasia; moreover, no abnormalities were palpable on the rectum wall, and no residual tumor or suspicious lymph nodes could be observed on MRI. A wall thickening of the rectum alone was not considered pathological.

TME is still the standard procedure for treating LARC after nRCT according to many guidelines (1, 2, 18) or some countries incorporate NOM into their guidelines (19). Many clinicians are compelled to perform TME even in the presence of cCR after nRCT, despite the known potential perioperative complications, morbidities and mortality as well as reduced quality of life (11, 12, 20, 21). Furthermore, cardiopulmonary and thromboembolic postoperative complications are independently associated with worse overall survival (22). In cases where individualized NOM with the W&W strategy is offered, no consensus on follow-up or surveillance exists in national guidelines to detect local regrowth or distant recurrence, unlike after TME. Ever since the pioneering work of Nakagawa et al. in 2002 and Habr-Gama et al. in 2004 (23, 24), the use of NOM for the treatment of rectal cancer has gained popularity worldwide. In recent reviews, no difference in overall survival or disease-free survival was found between patients treated with TME and patients managed with the W&W strategy (13, 25, 26). Under vigorous surveillance with early detection of local regrowth, a W&W strategy appears feasible and safe and allows a high rate of successful salvage surgery without increasing the risk of systemic disease or without compromising survival outcomes (25).

Local regrowth occurs mostly within 2 years after nRCT (27). Therefore, we decided to perform follow-up evaluations every 3 months for the first 3 years and thereafter every 6 months until the fifth year after initial diagnosis. The evaluations included digital rectal examination, rectoscopy, CEA measurements, chest radiology, abdominal ultrasound and pelvic MRI. If a lesion, e.g., in the liver, could be suspected or if elevated CEA levels could be measured, a CT scan of the abdomen would have been performed to rule out distant metastasis. Using this stringent follow-up schedule it was possible to monitor the patient appropriately without fear of missing out a local regrowth or distant recurrence. In addition, this approach increased patient satisfaction and reduced psychological distress, which is an aspect of quality of life. We agree fully with Huisman et al. that by using a structured follow-up in the case of cCR after nRCT, an organ-preserving NOM with the W&W strategy can be a safe procedure (28). In their study, they planned the first evaluation 8 weeks after completion of the nRCT and the second 12–16 weeks later. The evaluations in the W&W program included endoscopy, rectal MRI, abdominal and thoracic CT, and CEA screening every 3–6 months. Interestingly, they had a 3-year cumulative local regrowth incidence of 42%, and one patient was even censored out of the W&W program due to incurable distant recurrence after 5 months. This highlights the need for careful patient selection and reflects the persistent challenge of identifying complete responders as well as incomplete responders through a genuine surveillance strategy (29).

The management of LARC is continually progressing, and total neoadjuvant therapy (TNT) with NOM may become the standard of care for approximately one-third of patients in the future since responses to nRCT appear to be heterogeneous because of differences in immunological and genetic profiles (26, 30). Additionally, Chatila et al. reported compressively the clinical relevance of genomic and transcriptomic determinants such as insulin-like growth factor 2 (IGF2) and L1 cell adhesion molecule (L1CAM) (31). Overexpression IGF2 and L1CAM was associated with decreased response to neoadjuvant therapy and therefore correlates with poor outcomes in LARC. Furthermore, it has been shown that patients with high microsatellite instability tumors respond differently to neoadjuvant therapy compared to those with microsatellite stable tumors (32). Thus, patients with microsatellite instability tumors can benefit from immunotherapy and less from nRCT or TNT. The results of recent trials, e.g., the RAPIDO, PRODIGE 23, CAO/ARO/AIO-12 and OPRA trials (33–36), showed that NOM or WW strategies should be part of the treatment discussion for LARC. However, substantial evidence of long-term outcomes, including quality of life, is needed for patients with cCR managed by NOM via the W&W strategy after nRCT or TNT from multinational, prospective and randomized trials to formulate future guidelines. Furthermore, these trials should account for the challenges that clinicians face in real-world clinical assessment by identifying responders after nRCT or TNT treatment regimens.

In summary, based on our experience in a series of cCR cases, we recommend the following surveillance intervals for follow-up program; digital rectal examination, rectoscopy, CEA level measurements and pelvic MRI every 3–4 months in the first 2 years, and then once every 6 months until the fifth year after diagnosis. A chest and abdominal CT should be performed annually to rule out distant metastasis.

## Conclusion

We highlighted the use the W&W strategy in cases of cCR after an nRCT for rectal cancer with a structural follow-up program of more than 5 years. Through a genuine surveillance approach the W&W strategy does not seem to increase the risk of systemic disease or compromise survival outcomes in selected locally advanced rectal cancer patients. Nevertheless, for successful NOM with the W&W strategy, detailed patient information about the consistency of the follow-up program and patient compliance is mandatory. Thus embracing the need for personalized medicine in treatment discussion of locally advanced rectal cancer.

## Data Availability

The raw data supporting the conclusions of this article will be made available by the authors, without undue reservation.
